# The Chemoprevention Effects of Two Herbal Mixtures on Chemically Induced Lung Tumorigenesis in Mice

**DOI:** 10.3390/ph16121666

**Published:** 2023-11-30

**Authors:** Chunjie Li, Yian Wang, Song Gao, Ming Hu, Ming You

**Affiliations:** 1 Division of Medical Oncology, Department of Internal Medicine, The Ohio State University Comprehensive Cancer Center, Columbus, OH 43210, USA; chunjie.li@osumc.edu; 2 Center for Cancer Prevention, Houston Methodist Cancer Center, Houston Methodist Research Institute, Weill Cornell College of Medicine, Houston, TX 77030, USA; ywang8817c@houstonmethodist.org; 3 Department of Pharmaceutical Science, College of Pharmacy and Health Science, Texas Southern University, Houston, TX 77004, USA; 4 Department of Pharmacological and Pharmaceutical Science, College of Pharmacy, University of Houston, Houston, TX 77204, USA; mhu4@central.uh.edu

**Keywords:** Ruan Hua Tang, Ruan Hua Fang, chemoprevention, lung cancer, A/J mice

## Abstract

Ruan Hua Tang (RHT) and Ruan Hua Fang (RHF) are two Chinese herbal mixtures that have been used in clinical cancer treatment for decades. This study validated our hypothesis that RHT and RHF can inhibit lung tumor development in the mouse model of Benzo(a)pyrene-induced lung tumorigenesis. An RHT oral solution was diluted to 9% and 18% in water. RHF was mixed into the diet at 15% and 30% of total food in the final doses. Two weeks after injecting BP into mice, we administered RHT and RHF for eighteen weeks. We found that 9% and 18% RHT reduced tumor multiplicity by 36.05% and 38.81% (both *p* < 0.05) and the tumor load by 27.13% and 55.94% (*p* < 0.05); 15% and 30% RHF inhibited tumor multiplicity by 12.75% and 39.84% (*p* < 0.01) and the tumor load by 18.38% and 61.68% (*p* < 0.05). Ki67 expressions in the 9% and 18% RHT groups were 19.55% and 11.51%, significantly lower than in the control (33.64%). The Ki67 levels in the 15% and 30% RHF groups were 15.56% and 14.04%, significantly lower than in the control (27.86%). Caspase 3 expressions in the 9% and 18% RHT groups were 5.24% and 7.32%, significantly higher than in the control (2.39%). Caspase 3 levels in the 15% and 30% RHF groups were 6.53% and 4.74%, significantly higher than in the control (2.07%). The bio-absorption was confirmed via a pharmacokinetic test. This study showed that RHT and RHF are safe and can inhibit lung tumor development, with anti-proliferative and pro-apoptotic effects.

## 1. Introduction

Lung cancer is the leading cause of cancer death in the United States [1]. Tobacco smoking has been implicated in 90% of lung cancers, and smokers have a 20-fold higher risk of developing lung cancer than lifetime nonsmokers [2]. Although many novel treatments and medications have been developed in the past decade, e.g., immunotherapy and targeted therapy, lung cancer has not been completely eradicated. Prevention could be an ideal intervention to effectively decrease the incidence and mortality of lung cancer in both current and former smokers. Chemoprevention is the inhibition or reversal of the carcinogenic process through the use of drugs or other compounds [3] with the goals of either primary prevention to block the development of early lesions or secondary prevention to inhibit the evolution of early lesions into invasive cancers. Many agents, such as α-tocopherol, β-carotene [4], aspirin [4], retinol [5,6,7], isotretinoin [8], N-acetylcysteine [9], and anethole dithiolethione [10], have been investigated in clinical trials. Unfortunately, no benefit in preventing lung cancer was observed from those trials. Meanwhile, identifying agents with acceptable safety and efficacy profiles for human trials remains challenging [11,12].

Traditional Chinese medicine (TCM) has been widely used to prevent and treat cancers in China. In clinical practice, herbal mixtures and recipes are the most commonly used regimens in TCM. We previously assessed the chemopreventive effect of an herbal mixture, Antitumor B (ATB), on chemical-induced lung tumorigenesis animal models based on its success in clinical trials at preventing esophageal precancerous lesions [12,13,14]. Encouraged by these studies, we have proposed to further investigate the preventive efficacy of other Chinese herbal regimens. Ruan Hua Tang (RHT), a premade oral solution, is composed of *common selfheal fruit-spike*, *seaweed*, *oyster shell*, *jack-in-the-pulpit tuber*, *catclaw buttercup root*, *amorphophallus konjac*, *sun euphorbia herb*, *snakegourd peel* and *light-yellow Sophora root*. RHT has been clinically used for cancer treatment in China for over forty years. Ruan Hua Fang (RHF) is another Chinese herbal recipe composed of eight herbs, including *jack-in-the-pulpit tuber*, *common selfheal fruit-spike*, *seaweed*, *catclaw buttercup root*, *amorphophallus konjac*, *appendiculate cremastra pseudobulb*, *snakegourd peel*, and *dried tangerine peel*. RHF is an herbal mixture clinically used to treat cancers in China. Both Chinese herbal mixtures are safe for human use (ascertained through personal communication with Dr. Xu Ling, Long Hua Hospital, Shanghai, China).

In this study, we hypothesized that RHT and RHF have chemopreventive efficacies on lung cancer and that they could be candidates for further exploration in chemoprevention research. We have evaluated the effects of RHT and RHF using benzopyrene (BP)-induced lung tumorigenesis in A/J mice. We monitored lung tumor multiplicity, lung tumor load, and the expressions of Ki67 and caspase 3 in the tumor cells via immunochemistry (IHC). This study showed that RHT and RHF inhibit lung tumor growth through their anti-proliferative and pro-apoptotic effects. This is the first study to show that RHT and RHF can prevent lung tumor growth and to provide valuable candidates for use for lung cancer chemoprevention in humans.

## 2. Results

### 2.1. Inhibitory Efficacy of RHF and RHT on BP-Induced Lung Tumorigenesis

**Figure 1:**
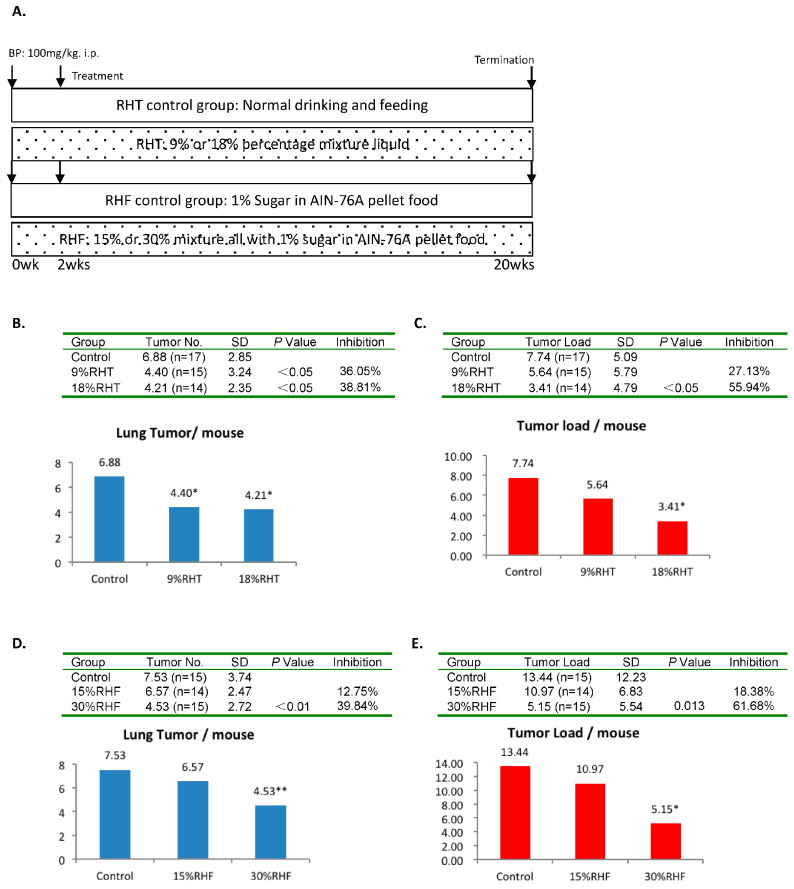
Effects of RHT and RHF on BP-induced lung tumorigenesis in A/J mice. (**A**) Experimental design. The upper panel shows the protocol for the RHT test, and the lower panel shows the protocol for the RHF test. All mice were intraperitoneally injected with a single dose of BP (100 mg/kg body weight). Two weeks later, RHT and RHF were administrated. The total duration of treatment was eighteen weeks. (**B**) The results of the RHT test on tumor multiplicity: 9% and 18% RHT significantly decreased tumor multiplicity by 36.05% and 38.81%, respectively, *p* < 0.05. (**C**) The results of the RHT test on tumor load: 9% and 18% RHT reduced tumor load by 27.13% (*p* > 0.05) and 55.94% (*p* > 0.05), respectively. (**D**) The results of the RHF test on tumor multiplicity: 15% and 30% RHF decreased tumor multiplicity by 12.75% (*p* > 0.05) and 39.84% (*p* < 0.01), respectively. (**E**) The results of the RHF test on tumor load: 15% and 30% RHF reduced tumor load by 18.38% (*p* > 0.05) and 61.68% (*p* < 0.05), respectively. * *p* < 0.05. ** *p* < 0.01.

**Figure 2:**
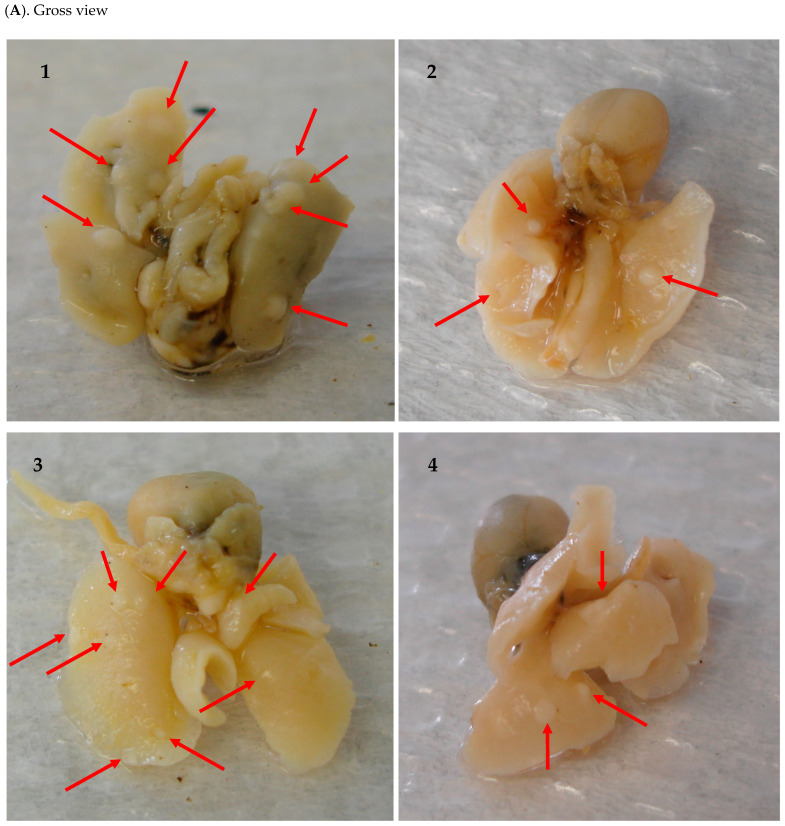

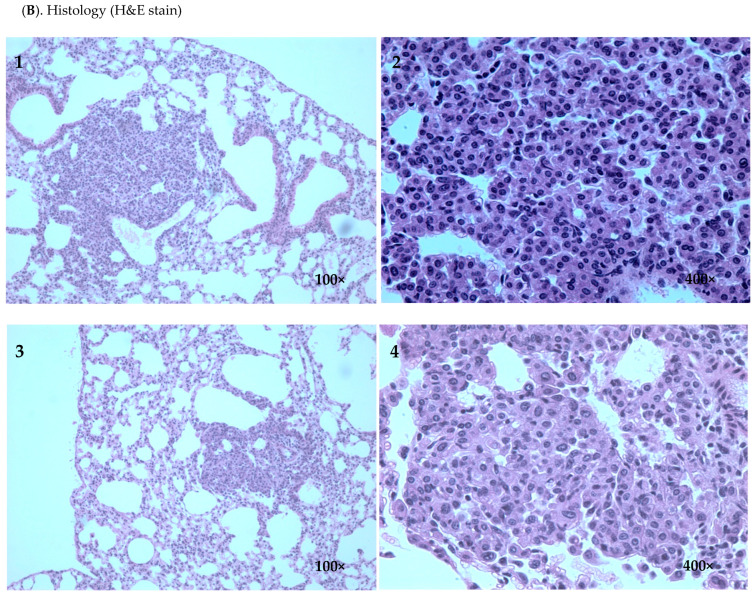
BP-induced pulmonary tumor lesions in A/J mice and their histology. After cutting the trachea, Tellyesniczky’s solution was injected through the trachea to perfuse both the left and right lungs, and then the entire lung was collected. (**A**) Gross view of lung nodules. Red arrows indicate tumors. (**A1**) RHT control group. (**A2**) RHT treatment group. (**A3**) RHF control group. (**A4**) RHF treatment group. The numbers of lung nodules in control groups were identifiably more than those in treatment groups. (**B**) H&E stain of tumor lesions and light photomicrographs of representative adenomas. (**B1**,**B2**) Control groups and (**B3**,**B4**) treatment groups, at 100× and 400× magnifications, respectively.

Two mice in the 9% RHT group and one mouse in the 30% RHF group did not have lung tumors by the end of the experiment. All other mice developed grossly identifiable lung tumors (Table 1). All mice treated with RHT and RHF showed no signs of gross toxicity or a loss of body weight throughout the experiment. The administration of 9% RHT significantly decreased tumor multiplicity (the average amount of tumor lesions per mouse) by 36.05% compared to the control group (*p* < 0.05). A significant 38.81% reduction in tumor multiplicity was also observed in the 18% RHT group compared to the control group (*p* < 0.05) (Table 2 and Figure 1B). Although 9% RHT showed an inhibition of the tumor load (the average accumulative volumes of all tumor lesions per mouse) of 27.13%, 18% RHT more significantly reduced the tumor load, by 55.94%, compared to the control group (*p* < 0.05) (Table 2 and Figure 1C). Similarly, 15% RHF decreased tumor multiplicity by 12.75% compared to the control group, while 30% RHF showed a more substantial inhibitory effect on tumor multiplicity of 39.84% compared to the control group (*p* < 0.01) (Table 3 and Figure 1D). The 15% RHF treatment reduced the tumor load by 18.38% compared to the control group. The 30% RHF treatment showed a 61.68% reduction in tumor load compared to the control group (*p* < 0.05) (Table 3 and Figure 1E). Overall, the higher doses of RHT and RHF more effectively inhibited tumor development. The tumor lesions on lung tissues are illustrated, via H&E staining, in Figure 2.

### 2.2. Tissue Concentrations of Rosmarinic Acid and Cucurbitacin B after Feeding Mice RHT or RHF

**Figure 3:**
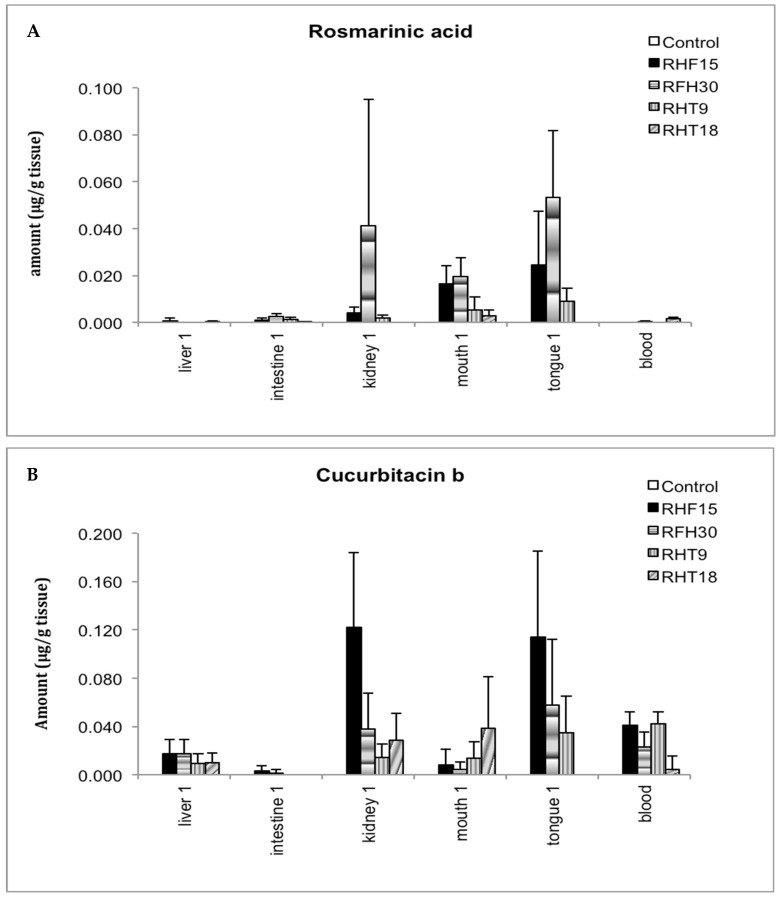
Tissue concentrations (μg/g tissue) of rosmarinic acid and cucurbitacin B in RHT- or RHF-treated tissues. (**A**) Tissue concentrations of rosmarinic acid. It was present in the tissues collected from mice treated with RHF or RHT with higher levels in the kidney, mouth, and tongue, but none was found in the control. (**B**) Tissue concentrations of cucurbitacin B. It was present in the tissues of mice treated with RHF or RHF with obviously higher levels in the kidney, mouth, tongue, and blood. Meanwhile, none was detected in control.

Rosmarinic acid and cucurbitacin B were selected for biodistribution studies after receiving the animal tissues. Both rosmarinic acid and cucurbitacin B were observed in the mice treated with RHF or RHT, while neither was detected in the control group. Specifically, rosmarinic acid was detected at high levels in the tongue and kidney, followed by cheek tissues, but at a low level in the small intestine. It was also detected in the blood samples of mice treated with 18% RHT. In addition, cucurbitacin B was found at a high concentration in the mouth cheek, tongue, blood, kidney, and liver tissues in all groups, while found at a relatively lower concentration in the small intestine (Figure 3).

### 2.3. Effects of RHF and RHT on Ki67 Expression

**Figure 4:**
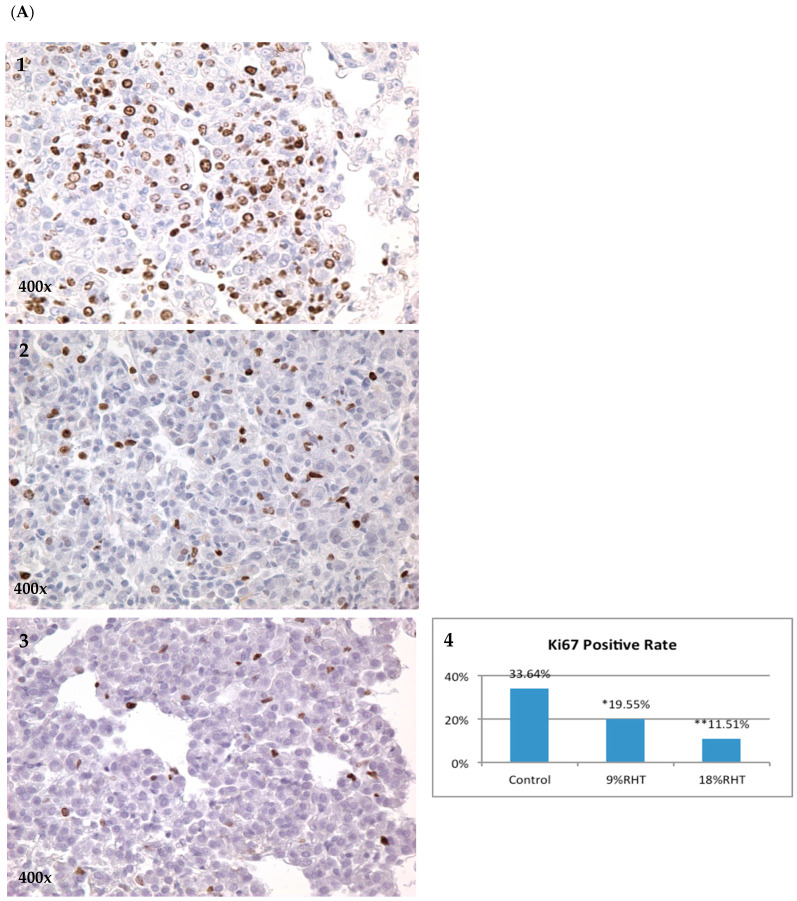

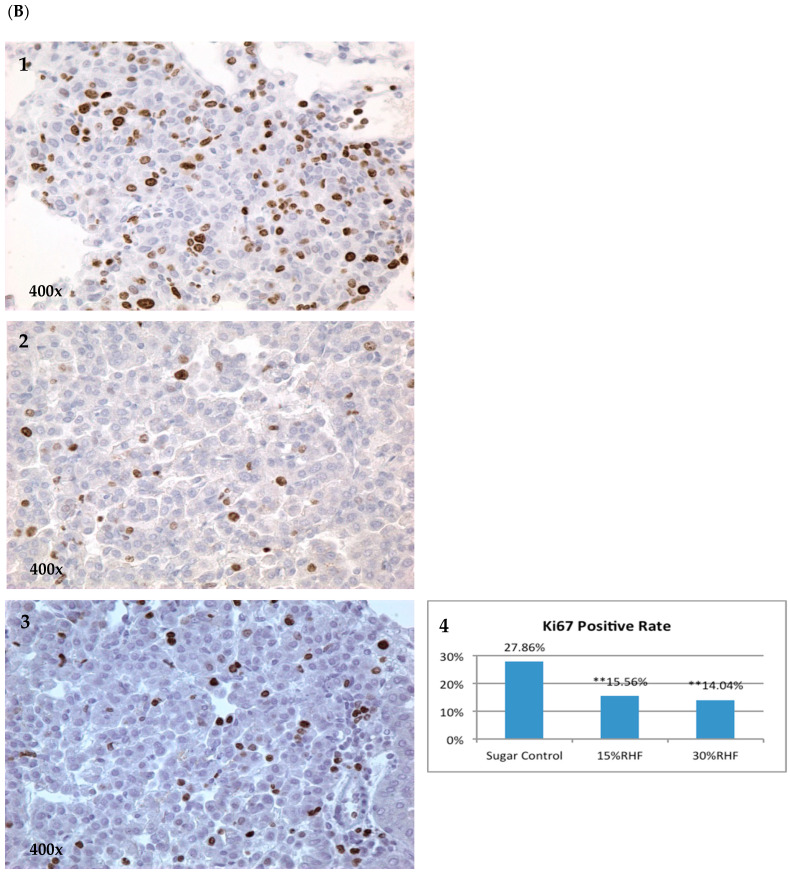
Ki67 expression in RHT and RHF groups via IHC. (**A**) Ki67 expression in RHT test: (**A1**) Control group. (**A2**) The 9% RHT group. (**A3**) The 18% RHT group. (**A4**) Both 9% and 18% RHT significantly inhibited Ki67 levels compared to the control group. (**B**) Ki67 expression in RHF test: (**B1**) Control group. (**B2**) The 15% RHF group. (**B3**) The 30% RHF group. (**B4**) Both 15% and 30% RHF significantly reduced Ki67 levels compared to the control group. * *p* < 0.05, ** *p* < 0.01.

Ki67 is a nuclear protein related to cellular proliferation, associated with ribosomal RNA transcription [15]. The inactivation of Ki67 leads to the inhibition of ribosomal RNA synthesis [16]. Ki67 can be used as a marker indicative of the level of tumor proliferation. In our study, Ki67 was measured via IHC in pulmonary tumor lesions with or without RHF/RHT treatments. The positivity of Ki67 in the 15% and 30% RHF groups were 15.56% and 14.04%, both of which were significantly lower than the 27.86% positivity in the control group (*p* < 0.05) (Figure 4B). The expressions of Ki67 were reduced by 44.15% and 49.61% in the 15% and 30% RHF groups, respectively. In the RHT experiment, the positivity of Ki67 in the 9% and 18% RHT groups were 19.55% and 11.51%, respectively, with a significant reduction by 41.88% and 65.78% compared to the control group (33.64%, both *p* < 0.05) (Figure 4A).

### 2.4. Effects of RHF and RHT on Caspase 3 Expression

**Figure 5:**
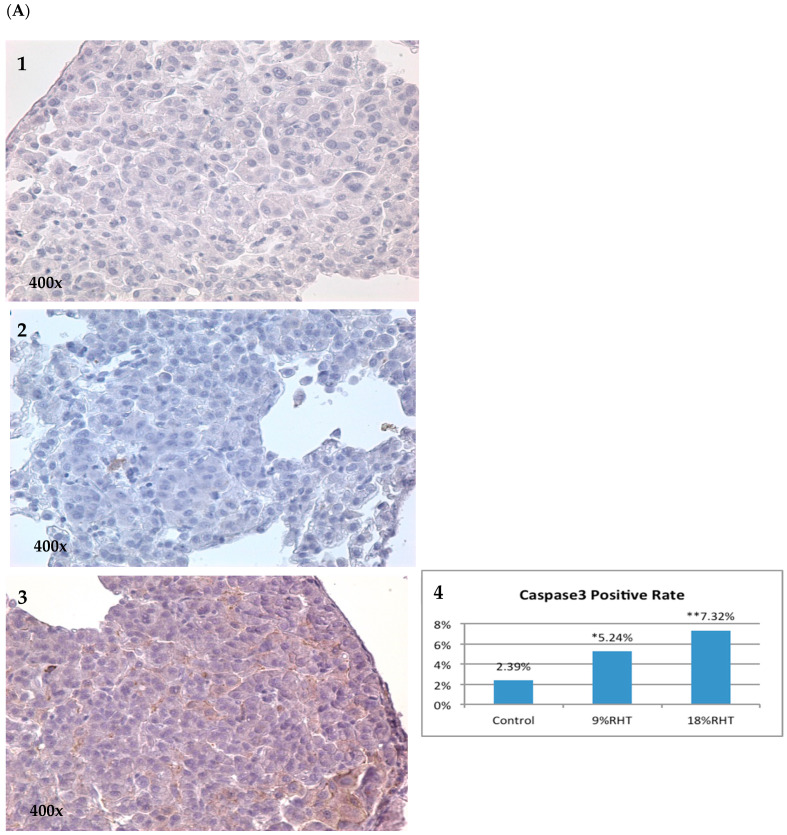

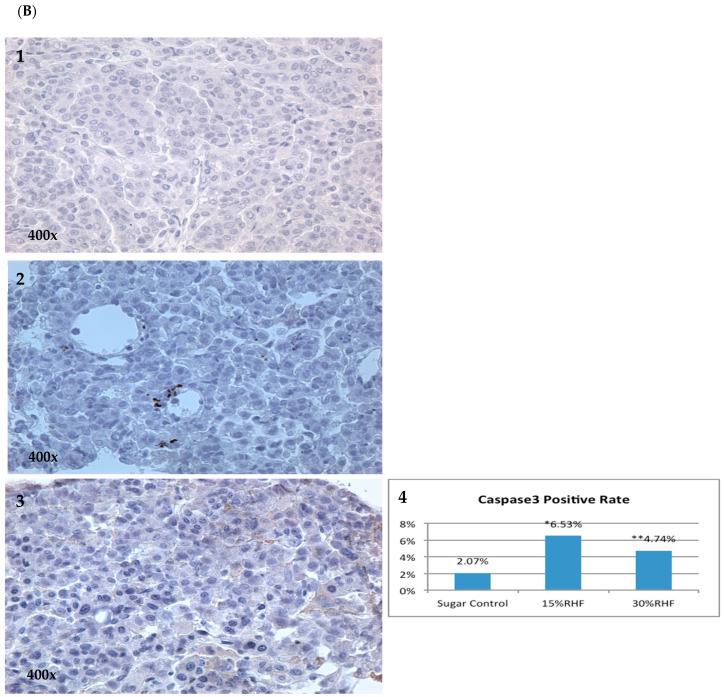
Cleaved caspase 3 (CC3) expression in RHT and RHF via IHC. (**A**) CC3 expression in RHT test: (**A1**) Control group. (**A2**) The 9% RHT group. (**A3**) The 18% RHT group. (**A4**) Both 9% and 18% RHT significantly increased CC3 expression compared to the control group. (**B**) CC3 expression in RHF test. (**B1**) The 1% sugar control group. (**B2**) The 15% RHF group. (**B3**) The 30% RHF group. (**B4**) Both 15% and 30% RHF significantly upregulated CC3 expression compared to the control group. * *p* < 0.05, ** *p* < 0.01.

Cleaved caspase 3 (CC3) is an essential mediator involved in apoptosis. To investigate whether RHF and RHT can promote apoptosis in tumor lesions, we quantified the expressions of CC3 via IHC in pulmonary tumor samples. We found that the positivity of CC3 in the 15% RHF and 30% RHF groups was 6.53% and 4.74%, respectively, significantly increased by 2.15-fold (*p* < 0.05) and 1.29-fold (*p* < 0.01) compared to the 2.07% positivity in the control group (Figure 5B). Similar results were seen in the RHT experiment; the positivity of CC3 in the 9% and 18% RHT groups were 5.24% and 7.32%, with a significant increase by 1.19-fold (*p* < 0.05) and 2.06-fold (*p* < 0.01) compared to 2.39% in the control group (Figure 5A).

## 3. Discussion

Traditional Chinese medicine (TCM) has been widely used to treat various cancers and some precancerous disorders in China. TCM compositions usually include multiple herbs and ingredients which contribute to their efficacy. Tremendous efforts have been made in expanding clinical trials to explore the survival benefits of TCM combined with chemotherapy and immunotherapy and elucidate the molecular mechanisms behind the therapeutic effects of herbal medicines. Many studies showed that TCM could regulate the proliferation, apoptosis, adhesion, and migration of cancer cells, inhibit tumor angiogenesis, change the host immune system, inhibit tumor growth, and modulate signaling transduction pathways to inhibit cancer growth and metastasis [17,18,19].

Given the promising progress and exploration of the therapeutic effects of Chinese herbal medicine used in cancer treatments, screening for effective herbal medicines that could potentially be candidates for chemoprevention is rational. ATB is one of the Chinese herbal regimens that has been used to treat esophageal precancerous lesions. We previously assessed the chemopreventive effects of ATB on two different mouse models and confirmed that ATB can inhibit the development of tumors induced by chemical carcinogens [12,14]. These previous studies and results have encouraged us to continue looking for more regimens from Chinese herbal medicine and validate their effects in terms of chemoprevention. RHT and RHF were clinically utilized in cancer treatment with a good reputation in Shanghai Longhua Hospital. Therefore, we decided to validate our hypothesis that these two Chinese herbal mixtures could inhibit the tumorigenic process.

In this study, we investigated the use of RHT and RHF on the chemoprevention of BP-induced lung tumors in the A/J mice model. Although most mice developed lung tumors in our study, three mice treated with RHT or RHF did not have lung tumors. More significantly, all mice that received RHT or RHF displayed a reduction in tumor multiplicity and the tumor load in their lung tumors, particularly in the groups receiving higher doses. Treatment with 9% or 18% RHT resulted in a 36.05% and 38.81% reduction in tumor multiplicity, with a 27.13% and 55.94% reduction in tumor load. Similarly, treatment with 15% or 30% RHF obtained a 12.75% and 39.84% reduction in tumor multiplicity and a 18.38% and 61.68% reduction in tumor load. These data illustrated the obvious inhibitory efficacies of RHF and RHT on the development of BP-induced lung tumors. Meanwhile, there was no gross toxicity nor loss of body weight observed in the study.

RHF and RHT share the same six herbs (*jack-in-the-pulpit tuber*, *common selfheal fruit-spike*, *seaweed*, *catclaw buttercup root*, *amorphophallus konjac* and *snakegourd peel*) in their recipes and have similar usage in clinical practice in China. The only difference is that RHT is a premade oral solution, while RHF is a mixed herbal combination that needs to be decocted. Our study also showed that RHT and RHF had similar inhibitory effects on the development of lung tumors in mice, indicative of their similar clinical usage. Furthermore, the same six herbal components in their recipes might be the active compounds responsible for their inhibitory effects. It suggests that further research on these herbs in the study of chemoprevention could eventually discover the most effective components in these herbal medicines.

Ki67 protein is a cellular marker for cellular proliferation [20]. During interphase, the Ki67 antigen can be exclusively detected within the cell nucleus, whereas in mitosis, most of the protein is relocated to the surface of the chromosomes. Ki67 protein is present during all active cell cycle phases (G1, S, G2, and mitosis) but is absent from resting cells (G0). The fraction of Ki-67-positive tumor cells (the Ki67 labeling index) is often correlated with the clinical course of cancer. Therefore, Ki67 is an excellent marker to determine the growth fraction of a given cell population. The caspase3 protein, interacting with caspase8 and caspase9 is a member of the cysteine-aspartic acid protease (caspase) family [21]. The sequential activation of caspases plays a central role in the execution phase of cell apoptosis. Caspases are inactive proenzymes that undergo proteolytic processing at conserved aspartic residues to produce large and small subunits that dimerize to form active enzymes. This protein cleaves and activates caspases 6 and 7, and the protein itself is processed and activated by caspases 8, 9, and 10. A recent study also found, under conditions of apoptotic induction or status, that activated caspase3 can cleave PAK2 (p58) to generate the active PAK2 form which can further promote apoptotic events [22]. Therefore, to confirm the anti-proliferative and pro-apoptotic efficacies of RHT and RHF in tumorigenesis, we measured Ki67 and CC3 expressions via IHC. The results revealed that Ki67 expressions in BP-induced lung adenomas were decreased, and CC3 expressions were increased after treating mice with RHF and RHT. It also suggested that RHT and RHF can inhibit the proliferation of tumor cells and promote apoptosis to interfere with the development of chemically induced lung tumors. These results also supported the description of tumor numbers and burden via gross observation.

In summary, our study confirmed that both RHT and RHF show significant inhibitory efficacies on BP-induced lung tumorigenesis in A/J mice by reducing both lung tumor multiplicity and tumor loads. Their actions might be associated with anti-proliferative and pro-apoptotic effects, given the reduction in Ki67 expressions and increase in CC3 expressions after RHT and RHF treatments. RHT and RHF were well tolerated and safe, without signs of long-term side effects or toxicities. These results suggested that RHT and RHF could potentially be used in future research on human lung tumor chemoprevention. Encouraging results from this study will thus set the path for future research on these Chinese herbal medicines for their use in chemoprevention.

While this experiment and these results are preliminary, they verify the effectiveness of RHT and RHF in inhibiting lung tumorigenesis. In future studies, we will test them on other mouse models to confirm the results from this study. The next step would also be to explore how RHT and RHF can regulate the signaling transduction pathways involved in lung tumor growth, e.g., the EGFR, Ras/Raf/MEK/ERK pathways, and the apoptotic pathways, e.g., Caspase/PAK2 and Bcl-2. These efforts could help elucidate the molecular mechanisms that allow them to interfere with the lung tumorigenic process.

## 4. Materials and Methods

### 4.1. Reagents and Animals

Both RHT premade oral solution and RHF herbal mixture (composed of jack-in-the-pulpit tuber 9 g, common selfheal fruit-spike 12 g, seaweed 9 g, catclaw buttercup root 15 g, amorphophallus konjac 15 g, appendiculate cremastra pseudobulb 9 g, snakegourd peel 15 g, and dried tangerine peel 9 g, for each recipe) were obtained from the department of pharmacy, Longhua Hospital, affiliated to the Shanghai University of TCM, Shanghai, China. Benzo(a)pyrene (BP) and tricaprylin were purchased from Sigma Chemical Co. (St. Louis, MO, USA). Eight-week-old A/J mice were purchased from Jackson Laboratory (Bar Harbor, ME, USA), fed, and maintained in the SIRF-West animal center at Washington University. The study was approved by Washington University’s Institutional Animal Care and Use Committee.

### 4.2. Animal Bioassays Using the BP-Induced Lung Tumorigenesis Model

The BP-induced lung tumor model in A/J mice was used in this study [14,23]. All mice were intraperitoneally injected with a single dose of BP (100 mg/Kg body weight) dissolved in 0.2 mL of tricaprylin two weeks before the treatment. Two weeks after BP injection, all mice were randomized into the following control and treatment groups: (1) control group with regular drinking and normal diet (AIN-76A purified pellet diet); (2) 9% RHT group drinking 9% RHT solution (*v*/*v*, diluted in purified water) and eating regular diet; (3) 18% RHT group drinking 18% RHT solution (*v*/*v*, diluted in purified water) and eating regular diet; (4) 1% sugar control group with 1% sugar mixed into AIN-76A purified pellet diet and regular drinking; (5) 15% RHF/1% sugar group (15% RHF group) with 15% RHF and 1% sugar (*wt*/*wt*) mixed into AIN-76A purified pellet diet and regular drinking; and (6) 30% RHF/1% sugar group (30% RHF group) with 30% RHF and 1% sugar (*wt*/*wt*) mixed into AIN-76A purified pellet diet and regular drinking. RHF herbal mixtures were ground into powder before they were mixed into the pellet diet. The results of the RHT experiments were compared between treatment groups and the control group, while the results of the RHF experiments were compared between treatment groups and the 1% sugar control group to tease out the possible confounding factor of 1% sugar in the diet.

All mice were sacrificed using CO_2_ asphyxiation 18 weeks after treatment, i.e., in the 20th week. Lung tissues were collected and immediately fixed in Tellyesniczky’s solution overnight, followed by storage in 75% ethanol. Two independent investigators assessed the number and size of pulmonary tumor lesions. They used a Leica MZ75 dissecting microscope to measure the number (N), volume (V), and total tumor load (N × V). Volume was calculated based on the formula: V (mm^3^) = 4/3πr^3^. Tumor load was determined according to the average total tumor volume per mouse. The inhibitory rate of the tumor number was calculated according to the formula: (average tumor number in control group—average tumor number in treatment groups)/average tumor number in control group × 100%. The inhibitory rate of the tumor load was determined according to the formula: (average tumor loads in the control group—average tumor loads in the treatment group)/average tumor loads in the control group × 100%.

### 4.3. Tissue Collection and Processing

Liver, small intestine, kidney, mouth cheek, tongue, and blood tissues or samples were collected in the 20th week after sacrificing the mice. All collected tissues were kept at −80 °C until use. Tissues were homogenized with 0.8 mL of IS in MeOH (0.5 µM, sulpiride) in a glass test tube, which was washed with 0.2 mL of MeOH twice. All the solution was combined, and 1.0 mL of supernatant was taken after centrifugation, which was air dried and reconstituted in 120 µL of MeOH for UPLC-MS analysis.

### 4.4. Immunohistochemical (IHC) Analysis

IHC reagent kits for the Ki67 and CC3 tests were purchased from Cell Signaling Technology. Briefly, slides were deparaffinized in xylene and rehydrated in gradient ethanol. Antigen retrieval was performed with citrate buffer (pH 6.0) in a microwave for 20 min. After blocking in 10% normal goat serum in PBS (0.1 mol/L sodium phosphate and 0.15 mol/L sodium chloride (pH 7.4)), the primary antibody was diluted in 10% normal goat serum and added to the slide, followed by incubation at 4 °C overnight. The secondary biotinylated IgG (1:500) was added to the slides the next day, followed by the avidin-biotin complex method (Vectastain ABC Elite kit, Vector Lab, Newark, CA, USA) according to the manufacturer’s protocol, with 3,3′-diaminobenzidine (Sigma) being used as the chromogen. For negative control, the primary antibody was omitted. Positively stained cells were determined by counting ten randomly chosen fields per section across the tumors.

### 4.5. Ultra-Performance Liquid Chromatography (UPLC) Conditions for Analysis

UPLC conditions for measurement analysis were: Waters Acquity™, PDA detector; column, Acquity UPLC BEH C18 column (50 × 2.1 mm ID 1.7 μM, Waters, Milford, MA, USA); mobile phase A, 100% water; mobile phase B, 100% Acetonitrile; gradient, 0–0.5 min—0% B, 0.5–1.0 min—0–20% B, 1.0–3.0 min—20–95% B, 3.0–3.5 min—95% B, 3.5–3.6 min—95–0% B, 3.6–4.0 min—0% B; flow, 0.50 mL/min; column temperature, 60 °C.

### 4.6. Mass Spectrometry (MS) Conditions for Analysis

The MS analysis was performed on an API 3200 Qtrap triple quadrupole mass spectrometer (Applied Biosystem/MDS SCIEX, Foster City, CA, USA) equipped with a Turbo Ionspray™ source. The quantification was performed using a multiple-reactions monitoring (MRM) method with ion pair transition to monitor each analyte. Unit mass resolution was set in both the mass-resolving quadruple Q1 and Q3, negative scan mode; ionspray voltage, −4.5 kV (5.5 kV for icariin); ion source temperature, 650 °C; nebulizer gas (gas 1), nitrogen, 50 psi; turbo gas (gas 2), nitrogen, 50 psi; curtain gas, nitrogen, 10. Compound-dependent parameters are in Table 4. For example, the MS/MS method for rosmarinic acid used the negative mode; ion spray voltage, −4.5 kV; ion source temperature, 650 °C; nebulizer gas (gas 1), nitrogen, 50 psi; turbo gas (gas 2), nitrogen, 50 psi; curtain gas, nitrogen, 10; declustering potential (DP), 96; collision cell end potential (CEP), 23; collision energy (CE), 49; collision cell exit potential (CXP), 2. The low detection limits (LLOQ) were 0.039 µM for rosmarinic acid and 0.078 µM for cucurbitacin B, respectively. Formononetin was used as the internal standard (I.S.).

### 4.7. Statistical Analysis

One-way ANOVA analysis was used to test the differences between the control group and two treatment groups. *LSD* test was used in the one-way ANOVA to test the difference between two groups among the three groups. The statistical analysis was calculated using SPSS16.0 software. Differences were determined using a two-tailed Student’s test. A *p* value of <0.05 was considered significant.

## 5. Conclusions

This was the first study to explore the inhibitory effects of both RHT and RHF on lung tumorigenesis and provided important references to support more extensive future research on herbal medicines in chemoprevention. Our study showed that both RHT and RHF can inhibit lung tumor development in the chemically induced lung tumor model in vivo. The changes in Ki67 and CC3 expressions suggested that this inhibition might be associated with anti-proliferative and pro-apoptotic effects. Pharmacokinetic experiments also confirmed that the mice absorbed the chemical components of RHT and RHF. Although the above results were encouraging for RHT and RHF’s ability to effectively inhibit tumorigenesis, this was a preliminary study, and further investigation is warranted. Future research on the regulation of the signaling transduction pathways involved in lung tumor growth, and the apoptotic pathways, will further elucidate the molecular mechanisms by which these two Chinese herbal medicines interfere with tumorigenesis.

## Figures and Tables

**Table 1:** The incidences of lung tumors.

Group	Number of Mice	Mice with Tumor	Incidence	Group	Number of Mice	Mice with Tumor	Incidence
Control	17	17	100%	Control	15	15	100%
9% RHT	15	13	87%	15% RHF	14	14	100%
18% RHT	14	14	100%	30% RHF	15	14	93%

**Table 2:** The impacts of RHT on tumor multiplicity and tumor load.

Group	Number of Tumors	SD	*p* Value	Inhibition	Group	Tumor Load	SD	*p* Value	Inhibition
Control	6.88 (n = 17)	2.85			Control	7.74 (n = 17)	5.09		
9%RHT	4.40 (n = 15)	3.24	<0.05	36.05%	9%RHT	5.64 (n = 15)	5.79		27.13%
18%RHT	4.21 (n = 14)	2.35	<0.05	38.81%	18%RHT	3.41 (n = 14)	4.79	<0.05	55.94%

**Table 3:** The impacts of RHF on tumor multiplicity and tumor load.

Group	Number of Tumors	SD	*p* Value	Inhibition	Group	Tumor Load	SD	*p* Value	Inhibition
Control	7.53 (n = 15)	3.74			Control	13.44 (n = 15)	12.23		
15%RHF	6.57 (n = 14)	2.47		12.75%	15%RHF	10.97 (n = 14)	6.83		18.38%
30%RHF	4.53 (n = 15)	2.72	<0.01	39.84%	30%RHF	5.15 (n = 15)	5.54	0.013	61.68%

**Table 4:** Compound-dependent parameters for UPLC-MS analysis.

Compound	Q1	Q3	DP	CEP	CE	CXP
Rosmarinic acid	359	161	−51	−26	−23	−1
Cucurbitacin b	557	497	−48	−27	−16	−3

## Data Availability

Data are contained within the article.
